# Effects of antioxidant intervention in patients with polycystic ovarian syndrome: A systematic review and meta-analysis

**DOI:** 10.1097/MD.0000000000030006

**Published:** 2022-08-12

**Authors:** Junde Zhao, Xiaohui Sui, Qingyu Shi, Dan Su, Zhiheng Lin

**Affiliations:** a Shandong University of Traditional Chinese Medicine, JinanShandong, China; b Shandong University of Finance and Economics, JinanShandong, China; c People’s Hospital of Lixia District of Jinan, Jinan, Shandong, China.

**Keywords:** antioxidant intervention, meta-analysis, polycystic ovary syndrome

## Abstract

**Background:**

The role of antioxidant intervention in polycystic ovary syndrome (PCOS) patients has been increasingly investigated in recent years. In order to further clarify whether antioxidant therapy is beneficial for PCOS patients and the emphasis of its effects, this study provides a systematic review and meta-analysis of randomized controlled trials examining the effect of antioxidant intervention on PCOS.

**Methods:**

Enrolled study designs related to antioxidant interventions and PCOS, published from 1999 to 2020, were searched from EMBASE, PubMed, and Web of Science databases to sort out proven studies on antioxidant interventions and PCOS. Data were reported as weighted mean difference (WMD) or standard mean difference with associated confidence intervals of 95%. The analysis was conducted using Stata version 16.0.

**Results:**

Twenty-three studies were included in total. Antioxidant intervention had a positive impact on homeostasis model assessment of insulin resistance (WMD = –0.37, *P* = .011) and Triglycerides (WMD = –25.51, *P* < .001). And antioxidant intervention did not improve testosterone levels significantly (WMD = –0.20, *P* = .2611). Subgroup analysis showed that except for the D-chiro-inosito subgroup, no difference in body mass index was observed between the intervention group and the control group.

**Conclusions:**

This meta-analysis demonstrates the efficacy of antioxidant intervention in patients with PCOS, demonstrating that antioxidant intervention has a significant effect on insulin resistance and lipid metabolism improvement. However, antioxidant intervention therapy has no discernible impact on testosterone levels or body mass index. Omega-3 may be a more effective antioxidant intervention for PCOS. In addition, this meta-analysis provides important reference opinions and treatment recommendations for PCOS.

## 1. Introduction

Polycystic ovary syndrome (PCOS) is a prevalent gynecological endocrine diseases that affects 5% to 20% of women of reproductive age globally.^[1–3]^ In addition to infertility and abnormal menstruation,^[4–6]^ patients also face economic burdens and long-term health risks.^[7,8]^ Numerous studies have demonstrated that PCOS patients tend to have insulin resistance.^[1,3,9–11]^

In addition, PCOS patients exhibit oxidative stress.^[12–14]^ In patients with PCOS, oxidative stress is closely associated with metabolic disorders, ovulation disorders, and difficulties in embryo transfer.^[2,4,15,16]^ This may be the reason why PCOS patients have an abnormal metabolic state and reduced fertility. Consequently, numerous studies are devoted to the treatment of polycystic ovaries by improving oxidative stress and have achieved a degree of curative effect.^[17,18]^ Antioxidants are a group of substances that help to capture and neutralize free radicals, thereby eliminating their damaging effects on the body. It may be helpful to treat PCOS with antioxidants or drugs that promote the antioxidant process in the body. Normally, the damaging effects of reactive oxygen species can be offset by a sophisticated antioxidant system, which involves enzymatic antioxidants, such as superoxide catalase, dismutase, paraoxonase, and peroxidase, as well as nonenzymatic antioxidant substances, such as thiols, glutathione, vitamin E, vitamin C, selenium, vitamin A, thioredoxin, and zinc.^[19]^

Several findings suggest that antioxidant intervention may improve insulin resistance and lipid metabolism in polycystic ovary syndrome. Nonetheless, there is no exhaustive and objective assessment of the effects of various antioxidant treatments on PCOS patients. To further investigate the viability of antioxidant interventions in the treatment of PCOS, we selected for meta-analysis all relevant studies involving 7 types of clinically common antioxidants.

## 2. Materials and Methods

### 2.1. Search strategy

A comprehensive literature search was conducted using PubMed, Web of Science, and EMBASE from their inception to March 2021 to identify all potentially relevant articles. All search methods utilized a systematic strategy in accordance with the Preferred Reporting Items for Systematic Review and Meta-Analysis Protocols.

The following terms were searched for: ((Coenzyme Q10) or (Inositol) or (Vitamin E) or (Selenium) or (Omega-3) or (Melatonin) or (zine)) and ((Polycystic Ovary Syndrome) or (Polycystic Ovarian) or (Syndrome, Polycystic Ovary) or (Ovary Syndrome, Polycystic)).

### 2.2. Selection criteria

Two reviewers independently performed a literature search, assessed potentially eligible studies for inclusion, and extracted data. Disagreements were resolved by negotiation with a third reviewer when necessary. If necessary, we contacted the authors of the original studies for extra info.

The primary criteria for inclusion are as follows:

1. Randomized controlled clinical trials. In the intervention group, patients received antioxidant treatment. In the control group, they got placebos or placebos plus the same drugs.
2. Study population: patients with PCOS. Two of the 3 diagnostic criteria for PCOS (oligoovulation and/or anovulation, clinical and/or biochemical signs of hyperandrogenemia, polycystic ovary) were recommended as the diagnostic criteria for PCOS.^[20]^
3. Aged 18 to 40 years.
4. Studies that report weighted mean difference (WMD) or standard mean difference (SMD) with corresponding 95% confidence intervals (CIs) or provide alternative methods to calculate or obtain these values.

### 2.3. Data extraction

Two researchers independently extracted data from eligible studies using this form and discussed discrepancies. Author, year of publication, age of patient, sample size, treatment method, WMD (95% CI) or SMD (95% CI), and variables controlled for matching or used in multivariable models were among the data collected. We entered the information into the software Review Manager (RevMan 5.3). According to the Cochrane scale’s quality standard, the Cochrane score was utilized to assess the quality of these selected studies. Two reviewers reconciled their differences through conversation. Disagreements were resolved, if necessary, through consultation with a third reviewer.

### 2.4. Data analysis

Six variables were extracted from each study as mean ± standard deviation. Our study utilized Stata version 16.0 to analyze data. *P* values are 2-sided, and a *P* value of < .05 was regarded as the statistical significance threshold. In addition, the heterogeneity of these 5 studies was evaluated. In this meta-analysis, we assessed the heterogeneity between the included studies using the *I^2^* statistic, with *I^2^* ≥ 50% indicating significant heterogeneity.^[21]^ For studies with *I²* ≥ 50%, we calculated using the random-effects model and for studies with *I²* < 50%, we used the fixed-effects model. With the 95% CI, the WMD or SMD for continuous variables was used to explain outcomes. We have also conducted in-depth research on subgroup (or regression) analysis and sensitivity analysis for some data with significant heterogeneity. In addition, the bias was described using Egger test method.

## 3. Results

### 3.1. Literature search and study characteristics

**Figure 1.**
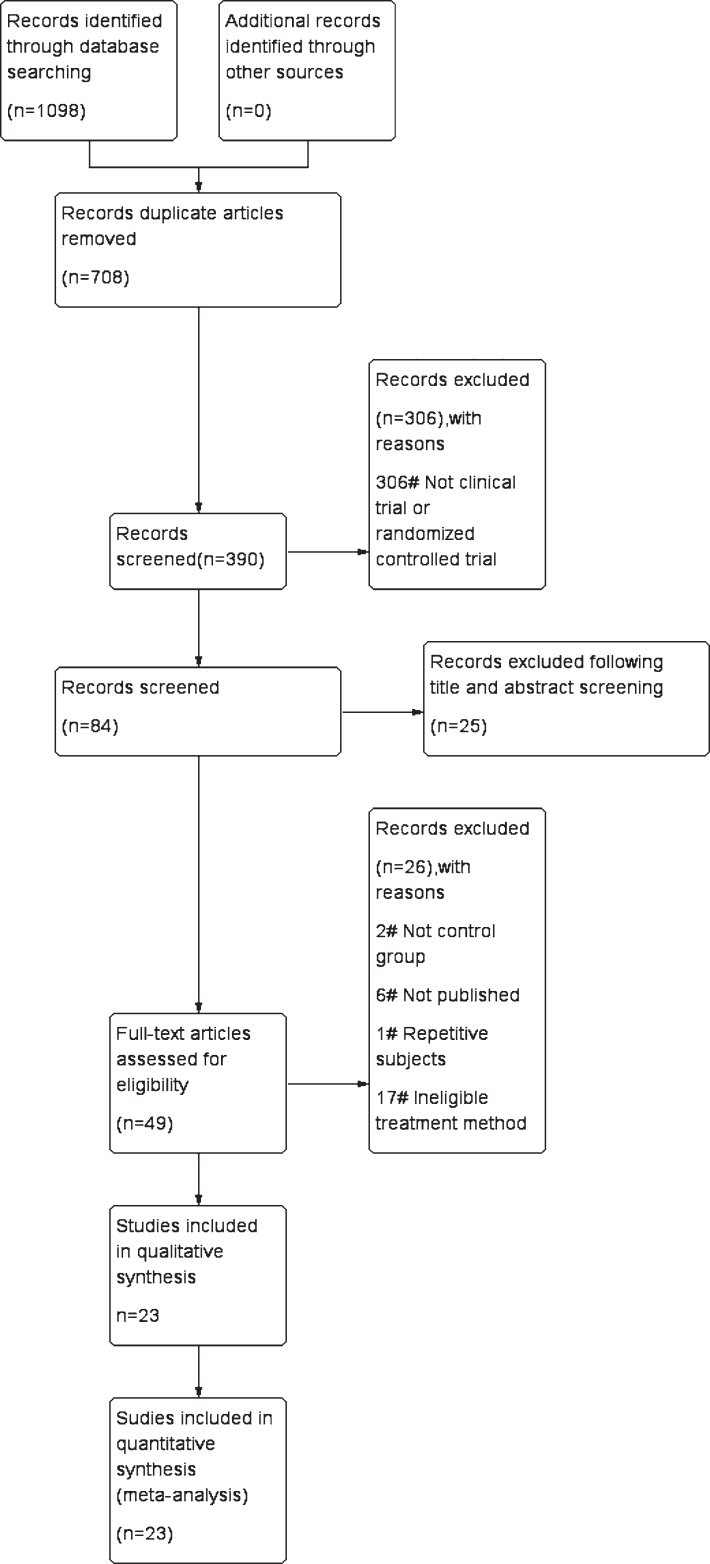
Flow diagram of study selection.

Figure 1 is a summary of the research selection process. The literature search yielded 1098 distinct references, of which 708 were considered duplicates. Three hundred six of these articles were omitted because they were of the incorrect inappropriate article type. Following title and abstract screening, 35 of these articles were excluded. The remaining 49, 26 were excluded due to sample characteristics (such as the absence of a control group) or a lack of pertinent data (e.g., not published). In total, 23 studies met the criteria for data extraction and inclusion in this meta-analysis. Table 1 provides a summary of their defining characteristics.

**Table 1:** The characteristics of included studies in this meta-analysis.

Author	year	Study type	Intervention	Sample	Duration of intervention
Izadi, et al^[22]^	2019	RCT	200 mg CoQ10 daily + vitamin E placebo\400 IU vitamin E daily + CoQ10 placebo\200 mg CoQ10 + 400 IU vitamin E daily\CoQ10 placebo + vitamin E placebo	22\22\21\21	8 wk
Jamilian et al^[23]^	2018	RCT	1000 mg omega-3 fatty acids plus 400 IU vitamin E supplements\placebo	20\20	12 wk
Mirmasoumi et al^[24]^	2018	RCT	1000 mg flaxseed oil omega-3 fatty acids\placebo	30\30	12 wk
Ebrahimi et al^[25]^	2017	RCT	1000 mg omega-3 fatty acids from flaxseed oil containing 400 mg α-linolenic acid plus 400 IU vitamin E supplements\placebo	34\34	12 wk
Rahmani et al^[26]^	2017	RCT	1000 mg omega-3 fatty acids from flaxseed oil containing 400 mg α-linolenic acid plus 400 IU vitamin E supplements\placebo	34\34	12 wk
Rahmani et al^[27]^	2018	RCT	100 mg CoQ10\placebo	20\20	12 wk
Samimi et al^[28]^	2017	RCT	100 mg CoQ10 supplements\placebo	30\30	12 wk
Shabani et al^[29]^	2019	RCT	10 mg melatonin\placebo	29\29	12 wk
Jamilian et al^[30]^	2018	RCT	8 × 10^9^ CFU/d probiotic plus 200μg/d selenium supplements\placebo	30\30	12 wk
Mohammad Hosseinzadeh et al^[31]^	2016	RCT	Daily administration of 200 g selenium\placebo	26\27	12 wk
Jamilian et al^[32]^	2015	RCT	200 µg/d selenium supplements\placebo	35\35	8 wk
Zadeh Modarres et al^[33]^	2018	RCT	200-μg selenium\placebo per day	20\20	8 wk
Heidar et al^[34]^	2020	RCT	200 μg/d of selenium\palcebo	18\18	8 wk
Rashidi et al^[35]^	2020	RCT	200 μg/d selenium\placebo	34\32	12 wk
Fruzzetti et al^[36]^	2017	RCT	Myo-inositol 4 g plus folic acid 400 mcg/d\placebo	24\30	6 mo
Nestler et al^[37]^	1999	RCT	The oral administration of 1200 mg of D-chiro-inositol\placebo once daily	22\22	6 wk
Iuorno et al^[38]^	2002	RCT	Initiated with either 600 mg of D-chiro-inositol\placebo once daily	10\10	6–8 wk
Jamilian et al^[39]^	2018	RCT	50,000 IU vitamin D every 2 wk plus 2000 mg/day omega-3 fatty acid from fish oil\placebo	30\30	12 wk
Shokrpour et al^[40]^	2019	RCT	250 mg/d magnesium plus 400 mg/d vitamin E supplements\placebo	30\30	12 wk
Jamilian et al^[41]^	2019	RCT	Participants were randomly divided into 2 groups to receive 250 mg/d magnesium plus 400 mg/d vitamin E supplements\placebo	30\30	12 wk
Hager et al^[42]^	2019	RCT	Assigned to either the “multinutrient supplementation group” (1 unlabeled soft capsule containing omega-3 fatty acids and 1 unlabeled tablet containing folic acid, selenium, vitamin E, catechin, glycyrrhizin, and Co Q10) or the “control group” (2 unlabeled soft capsules containing 200 μg folic acid each)	30\30	3 mo
Maktabi et al^[43]^	2018	RCT	Treated with 100 mg magnesium, 4 mg zinc, 400 mg calcium plus 200 IU vitamin D supplements (n = 30)\placebo (n = 30) twice a day	30\30	12 wk
Foroozanfard et al^[44]^	2015	RCT	220 mg zinc sulfate (containing 50 mg zinc) supplements\placebo per day	26\26	8 wk

### 3.2. Risk of bias in all studies

**Figure 2.**
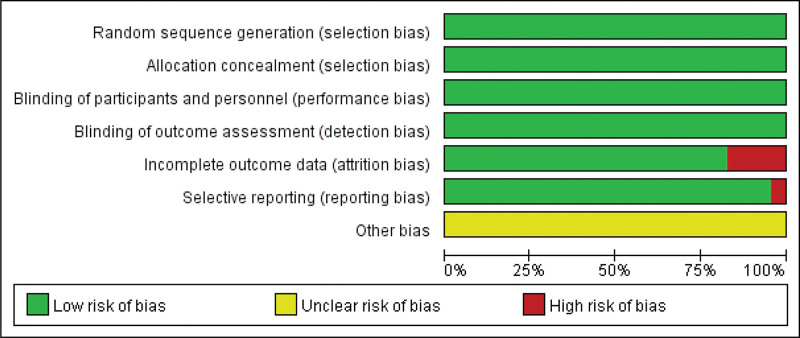
Summary of risk for each included study. Green means low risk of bias; yellow means unclear risk of bias; red means high risk of bias.

The risk of bias was selected for each randomized and prospective nonrandomized clinical study which was selected, the risk of bias was assessed according to the criteria described in the Cochrane Reviewers Handbook.^[27]^ The summary of the risk of bias is shown in Figure 2. All included studies were at low risk of bias level in terms of “selection bias”, “performance bias” and “detection bias.” Few of the included studies were at high risk of bias level in terms of “attrition bias” and “reporting bias.” The included studies were at unclear risk of bias level in terms of “Other bias.”

### 3.3. Effects on HOMA-IR

**Figure 3.**
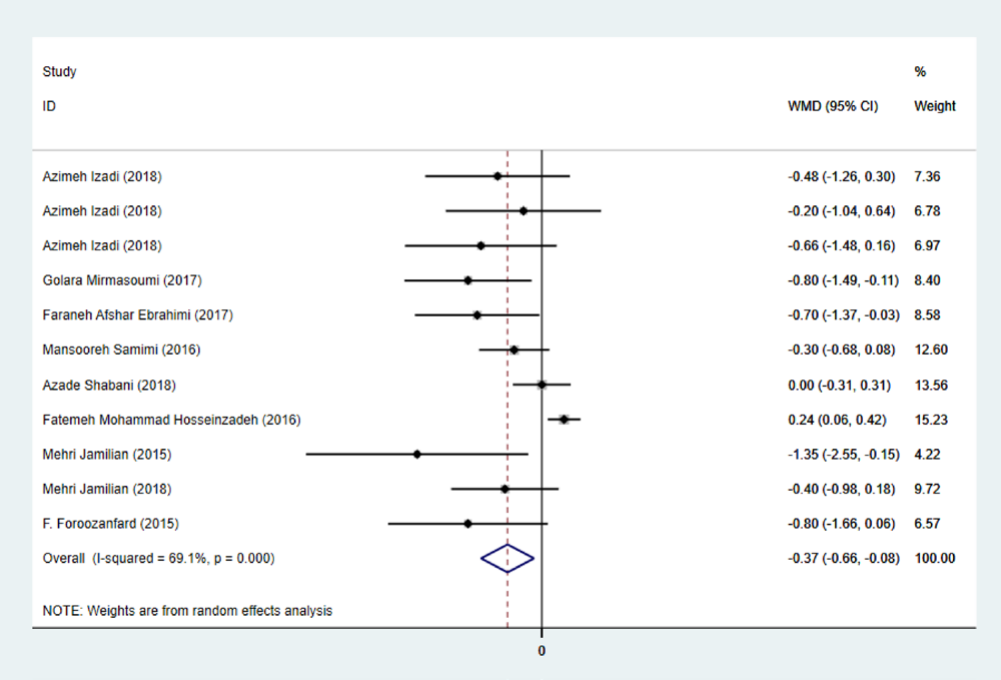
Forest plots of antioxidant intervention on HOMA-IR in patients with PCOS. HOMA-IR = Homeostasis Model Assessment of Insulin Resistance, PCOS = polycystic ovary syndrome.

Nine studies have addressed HOMA-IR indicators. HOMA-IR has been reported in eleven studies involving 609 subjects. There are 305 patients in the intervention group and 304 in the control group. Using a random-effects model, the WMD was 0.37 (95% CI –0.66 to –0.08, *P* = .011) lower in the intervention group than in the control group. Significant heterogeneity was observed (*P* = .000, *I*² = 69.1%; Fig. 3). This suggests that antioxidant treatment can reduce HOMA-IR significantly.

### 3.4. Effects on triglycerides

**Figure 4.**
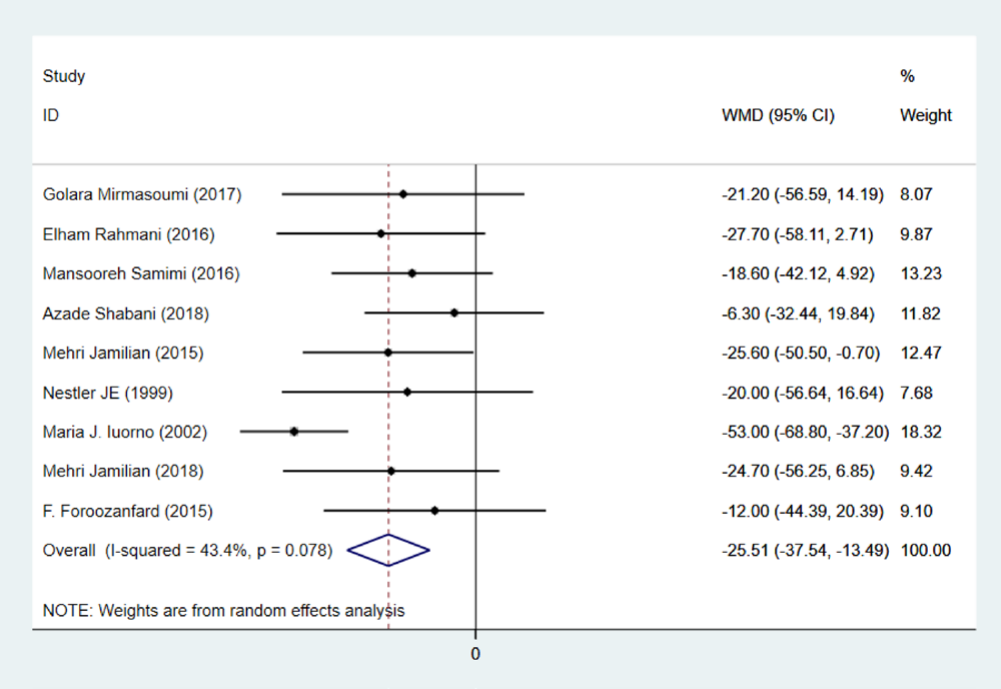
Forest plots of antioxidant intervention on triglycerides in patients with PCOS. CI = confidence interval, PCOS = polycystic ovary syndrome.

There are 9 studies on triglyceride indicators. Triglycerides were reported in 9 studies involving 492 subjects. There are 246 patients in the intervention group and 246 patients in the control group. Using a random-effects model, the WMD was 25.51 mg/dL (95% CI –37.54 to –13.49, *P* < .001) lower in the intervention group than in the control group. Heterogeneity was deemed insignificant (*P* = .078, *I*² = 43.4%; Fig. 4). This indicates that antioxidant therapy can significantly lower triglycerides.

### 3.5. Effects on testosterone

**Figure 5.**
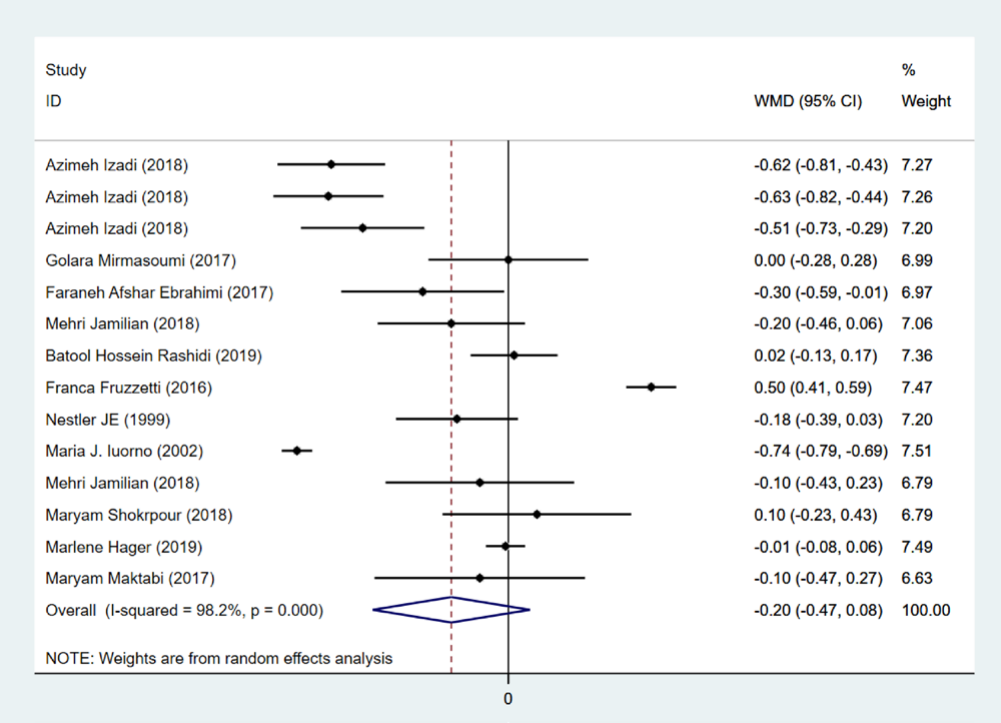
Forest plots of antioxidant intervention on testosterone in patients with PCOS. CI = confidence interval, PCOS = polycystic ovary syndrome.

Twelve studies have examined testosterone indicators. In 9 studies with 740 subjects, testosterone was reported. Three hundred sixty-nine patients are assigned to the intervention group and 371 to the control group. The current meta-analysis revealed that there was no difference in testosterone levels between intervention and control groups (WMD = –0.20 ng/mL, 95% CI –0.47 to 0.08, *P* = .2611). Figure 5 demonstrates that heterogeneity was considered significant (*P* < .001, *I*² = 98.2%), so a random-effects model was employed.

### 3.6. Effects on BMI

**Figure 6.**
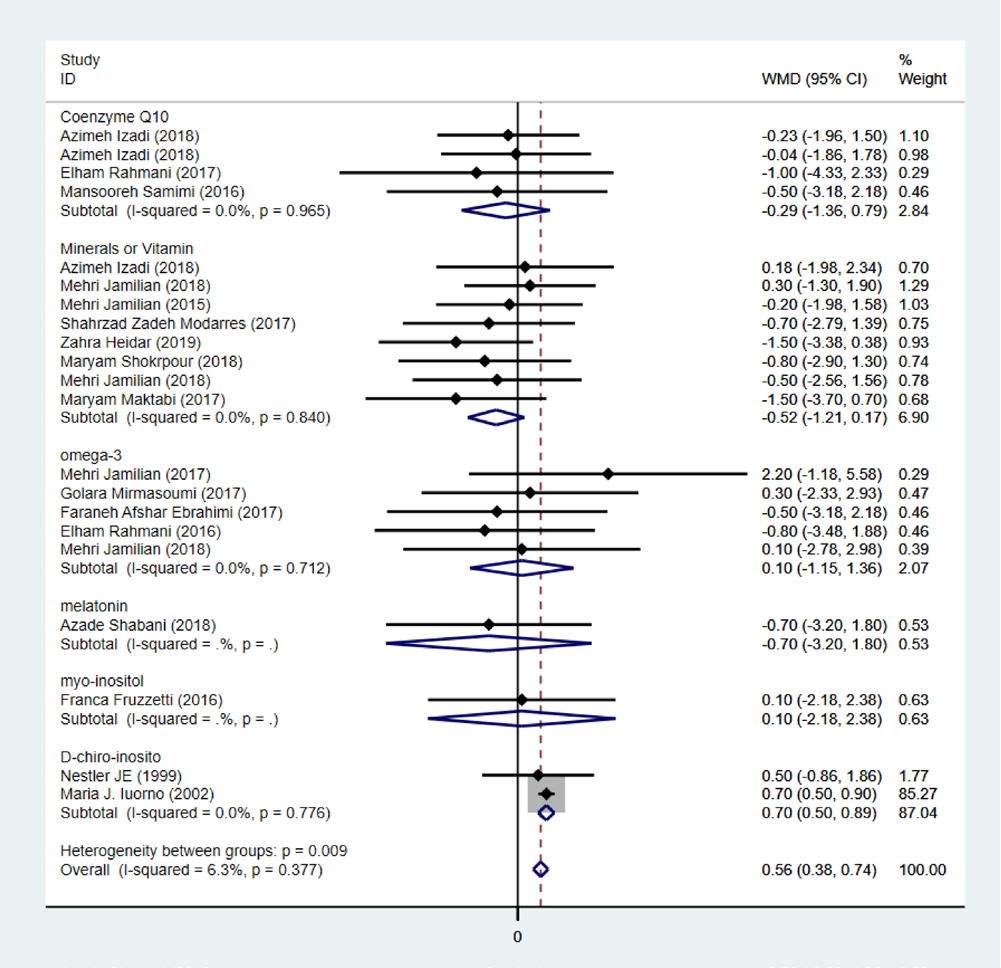
Forest plots of antioxidant intervention on BMI in patients with PCOS. BMI = body mass index, CI = confidence interval, PCOS = polycystic ovary syndrome.

Nineteen studies have addressed BMI indicators. BMI was reported in 9 studies involving 1086 subjects. Five hundred forty-one patients are assigned to the intervention group and 545 to the control group. The results of the meta-analysis showed that the BMI index increased in the antioxidant intervention group compared with the control group. However, this current meta-analysis showed no difference in BMI was witnessed between the intervention group and control group except the D-chiro-inosito subgroup. Heterogeneity was considered nonsignificant (Fig. 6).

### 3.7. Subgroup analysis of antioxidant intervention on HOMA-IR

By subgroup analysis, it was seen that although the antioxidant effect had an overall effect on HOMA-IR in PCOS patients, minerals or vitamin and melatonin did not have a significant effect on reducing HOMA-IR in patients with polycystic ovary syndrome. In contrast, both coenzyme Q10 and omega-3 had a significant effect on the reduction of HOMA-IR.

### 3.8. Subgroup analysis of antioxidant intervention on triglycerides

By subgroup analysis, it was seen that although the antioxidant effect had an overall effect on triglycerides in PCOS patients, coenzyme Q10 and melatonin did not have a significant effect on reducing triglycerides in patients with polycystic ovary syndrome. In contrast, both minerals or vitamin, D-chiro-inosito and omega-3 had a significant effect on the reduction of triglycerides.

### 3.9. Subgroup analysis of antioxidant intervention on testosterone

By subgroup analysis, it was seen that although the antioxidant effect had no effect on testosterone in PCOS patients. Minerals or vitamin, D-chiro-inosito and omega-3 did not have a significant effect on reducing testosterone in patients with polycystic ovary syndrome. In contrast, both coenzyme Q10 had a significant effect on the reduction of testosterone. Myo-inositol had a significant effect on the elevation of testosterone.

## 4. Discussion

In conclusion, the antioxidant intervention significantly improved insulin resistance and abnormal lipid metabolism in PCOS patients, but had no significant effect on testosterone, although the effect of the BMI analysis needs to be further investigated due to bias.

We have therefore conducted an appropriate subgroup analysis. The results of the subgroup analysis will have a profound effect on our ability to further differentiate the effects of different antioxidants on PCOS.

**Figure 7.**
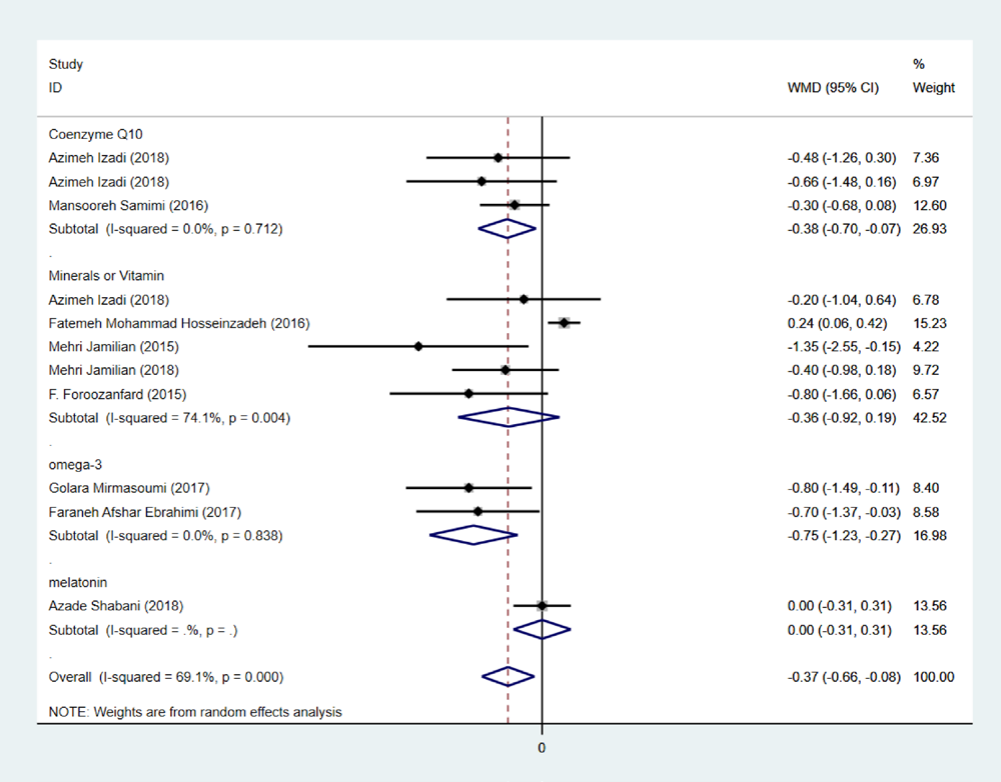
Subgroup analysis of antioxidant intervention on HOMA-IR in patients with PCOS. CI = confidence interval, HOMA-IR = Homeostasis Model Assessment of Insulin Resistance, PCOS = polycystic ovary syndrome.

In light of the insulin resistance of PCOS patients, we chose to analyze the HOMA-IR index, which is essential for measuring insulin resistance. This meta-analysis revealed that the intervention group had significantly lower HOMA-IR levels than the control group. This suggests that antioxidant therapy can significantly improve insulin resistance in PCOS patients. Certain evidence suggests that insulin resistance develops as a result of coordinated interactions between stress responses and various cellular stresses.^[45]^ Insulin resistance is associated with a specific increase in mitochondrial hydroperoxides, as determined by analyzing the oligomeric status of comparator-specific peroxiredoxins.^[46]^ Subgroup analysis revealed additional distinctions regulator within the group (Fig. 7). The coenzyme Q10 subgroup and the omega subgroup had significantly lower HOMA-IR levels than the placebo group. The *P* value of the HOMA-IR was not significantly different between the melatonin group and the nutrient element group. Previous research has demonstrated that coenzyme Q10 is a regulator of insulin and adiponectin receptors, phosphatidylinositol kinase 3, tyrosine kinase, and glucose transporters, suggesting that the antioxidant can enhance insulin sensitivity.^[47]^ Omega-3 fatty acids may improve insulin sensitivity by inhibiting proinflammatory mediators and decreasing nuclear factor-kappa B activation, thereby reversing insulin resistance.^[48]^

**Figure 8.**
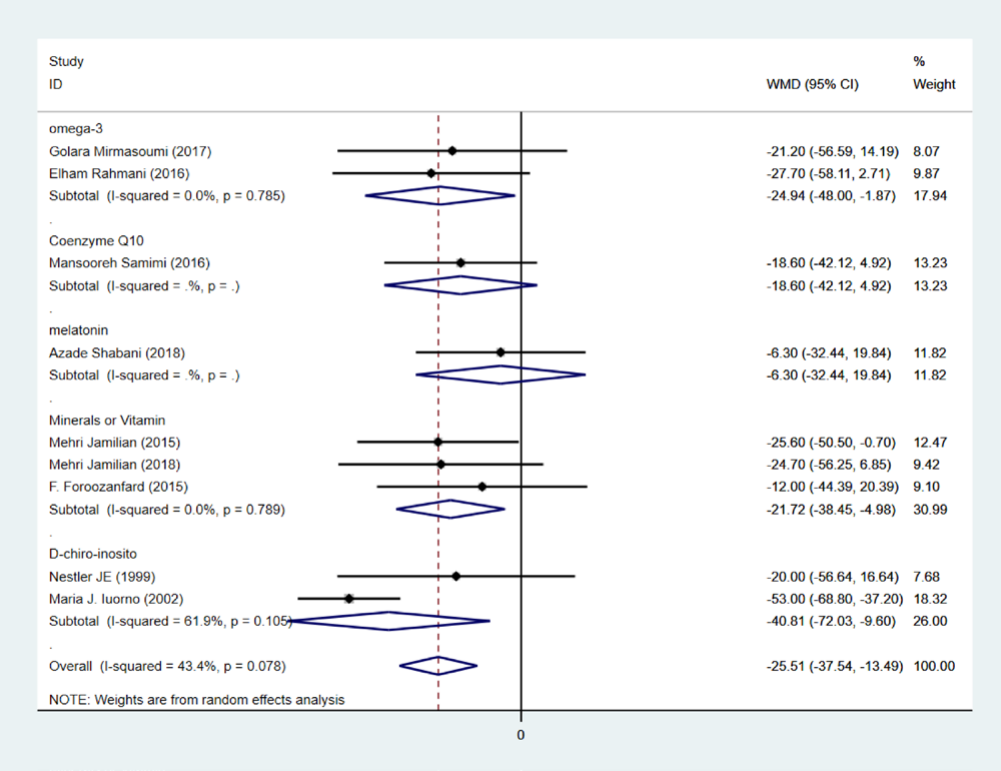
Subgroup analysis of antioxidant intervention on triglycerides in patients with PCOS. CI = confidence interval, PCOS = polycystic ovary syndrome.

In terms of lipid metabolism, this study revealed that the intervention group had significantly lower triglyceride levels than the control group. It indicates that the antioxidant treatment improves the lipid metabolism of PCOS patients significantly. Numerous previous studies have demonstrated this.^[24,26]^ Through regulating endoplasmic reticulum stress-related genes and cellular defense regulation reactive oxygen species, antioxidant therapy can decrease oxidative stress.^[49]^ However, we have subcategorized the various interventions. Subgroup analysis revealed that there was no significant difference in triglyceride levels between the melatonin and coenzyme Q10 subgroups (Fig. 8). It is noteworthy that the antioxidant effect on lipogenesis was comparable regardless of the antioxidant type. We cannot make accurate judgments because the number of research articles on the melatonin and coenzyme Q10 subgroups is insufficient.

**Figure 9.**
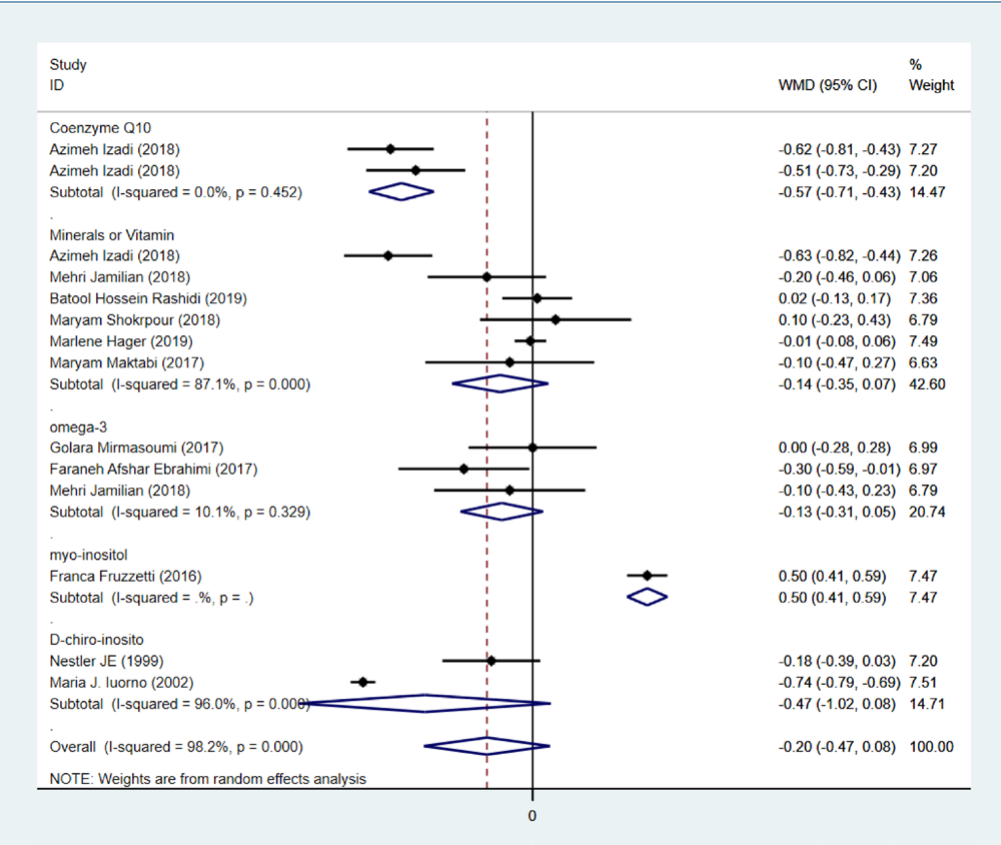
Subgroup analysis of antioxidant intervention on testosterone in patients with PCOS. CI = confidence interval, PCOS = polycystic ovary syndrome.

Figure 5 indicates that antioxidant intervention had no significant effect on testosterone improvement. Due to the significant heterogeneity, a subgroup analysis was conducted. According to Figure 9, coenzyme Q10 subgroup treatment significantly increases testosterone levels. The number of articles on indicators is relatively low, so we adopt a conservative stance. The current meta-analysis revealed no difference in BMI between the intervention and control groups, with the exception of the D-chiro-inosito subgroup. We speculate that it may be related to the duration of medication and the patients’ lifestyles. Nevertheless, it cannot be ruled out that this might be related to the short duration of the medication and the insufficient intervention in patients’ lifestyle.

**Figure 10.**
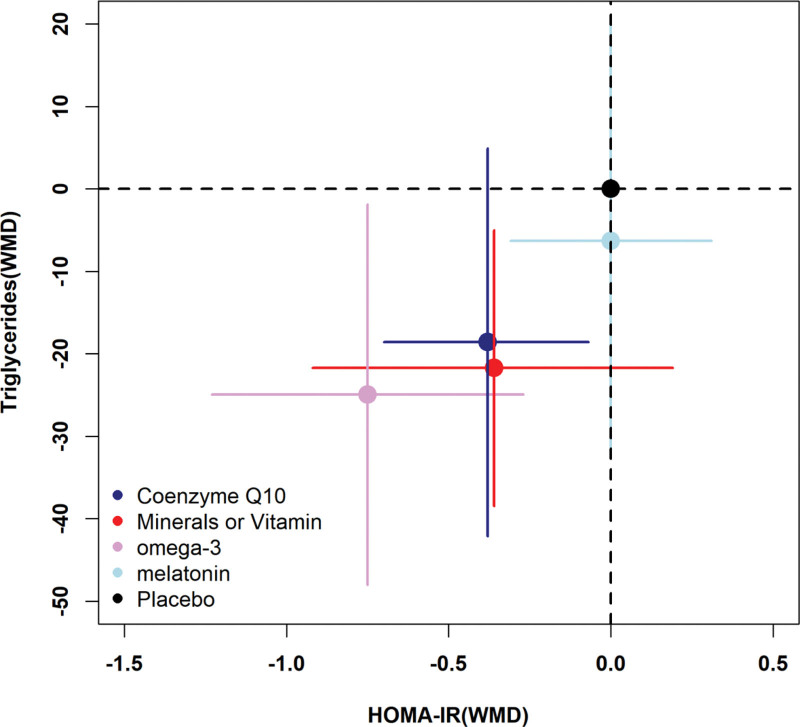
Two-dimensional plot on HOMA-IR and triglycerides. HOMA-IR = Homeostasis Model Assessment of Insulin Resistance.

In conclusion, the HOMA-IR and triglyceride indicators of PCOS patients can be significantly improved by antioxidant treatment. Consequently, the results of each subgroup are depicted using a 2-dimensional plot. According to Figure 10, omega-3 has a greater effect on the improvement of HOMA-IR and lipid metabolism overall. Numerous studies have demonstrated that omega-3 significantly reduces serum triglyceride levels in PCOS patients.^[50,51]^ It seems that an increase in adiponectin levels after supplementation with omega-3 fatty acids, which have antiatherosclerotic, anti-inflammatory, and antidiabetic effects, might improve insulin sensitivity.^[52]^ In muscles, adiponectin stimulates AMPK, which leads to downstream oxidative pathways.^[53]^ By activating the AMPK pathway, insulin control and blood lipid profiles are improved.^[54]^

When examining the results of this meta-analysis, certain limitations should be considered. The low quality obtained for HOMA-IR, triglycerides, and BMI was due to publication bias, as indicated by their Egger test *P* values of .000, .016, and .000.

Polycystic ovaries treatment requires a precise determination of the therapeutic effect of antioxidant intervention in PCOS patients despite our limitations. Different antioxidants have different effects on PCOS patients. As an antioxidant, omega-3 can reduce HOMA-IR, testosterone, and TG, and its potential therapeutic value for PCOS patients warrants further investigation. We propose that additional randomize controlled clinical trials of different antioxidant doses, medication duration, or drug combinations are required to demonstrate the therapeutic efficacy of antioxidant intervention in PCOS patients.

## 5. Conclusion

This meta-analysis demonstrates the efficacy of antioxidant intervention in patients with PCOS, demonstrating that antioxidant intervention has a significant effect on insulin resistance and lipid metabolism improvement. Omega-3 may be a more effective antioxidant intervention for PCOS.

## Author contributions

Junde Zhao: Data collection or management, Data analysis, Manuscript writing

Xiaohui Sui: Data collection or management, Data analysis

Qingyu shi: Data analysis, Manuscript writing

Dan Su: Data analysis, Manuscript writing

Zhiheng Lin: project development
